# Application of Biological Invasion Models in Epidemiology: Analysing the Spatial Distribution of Dengue Fever Cases Based on the Suitable Habitats of Aedes Mosquitoes

**DOI:** 10.1049/syb2.70087

**Published:** 2026-11-13

**Authors:** Cheng Li, Fang Lan, Yongbin Zhou, Jie Ji, Ji Liu

**Affiliations:** ^1^ School of Finance and Economics Shenzhen University of Information Technology Shenzhen China; ^2^ Shenzhen Customs District P.R. China Shenzhen China; ^3^ School of Foreign Languages Shenzhen University of Information Technology Shenzhen China; ^4^ School of Electrical and Computer Engineering The University of Sydney Sydney Australia

**Keywords:** biological techniques, biomedical engineering, biotechnology, diseases, epidemics

## Abstract

This study explores the application of biological invasion models in epidemiology by examining the spatial relationship between dengue fever transmission and the suitable habitats of *Aedes aegypti* and *Aedes albopictus* in China. By integrating invasion biology and epidemiological modelling perspectives, the study applies the MaxEnt model to predict mosquito suitable habitats using occurrence records and environmental variables, and further combines the spatial Durbin model to analyses the direct and indirect spatial effects of mosquito distribution on dengue incidence. Results reveal significant spatial correlations between mosquito habitats and dengue outbreak regions. *A. aegypti* exhibits stronger direct effects on local dengue transmission, suggesting higher infectivity within suitable habitats, whereas *A. albopictus* demonstrates stronger spatial spillover effects, indicating greater diffusion capacity across regions. The findings highlight the value of integrating species distribution modelling with spatial econometric analysis to improve understanding of vector‐borne disease diffusion and support targeted prevention and control strategies from an interdisciplinary systems perspective.

## Graphical abstract

This study investigates the application of biological invasion models in epidemiology by integrating the MaxEnt model and spatial Durbin model to analyse the spatial relationship between Aedes mosquito habitats and dengue fever transmission in China. Results demonstrate significant spatial correlations between mosquito suitable habitats and dengue outbreaks, revealing that *Aedes aegypti* has stronger local transmission effects whereas *Aedes albopictus* exhibits greater spatial diffusion capacity. The findings provide a systems‐level modelling framework for understanding vector‐borne disease diffusion and supporting targeted prevention strategies.

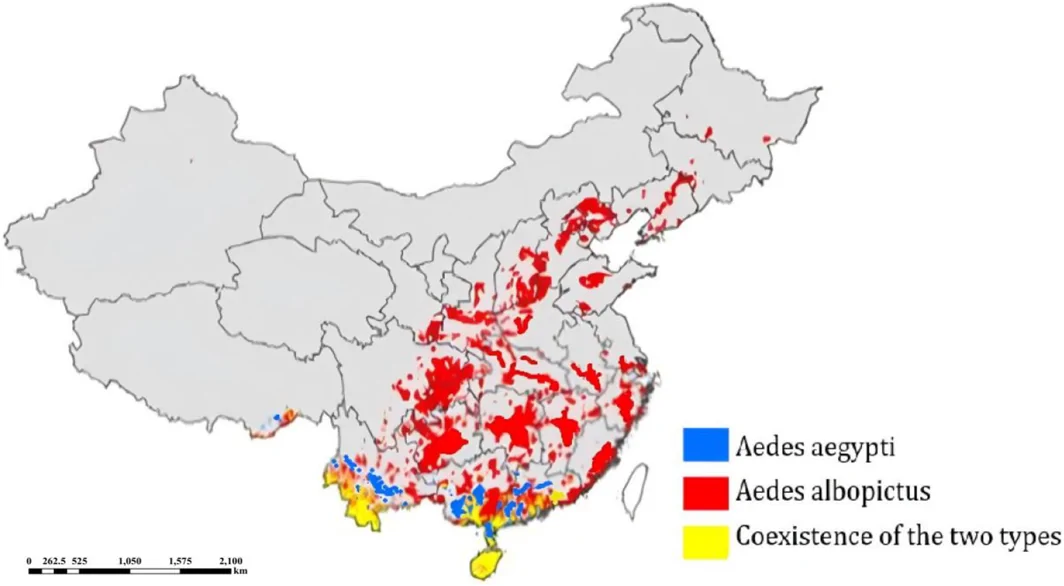

## 1 Background

With the advancement of globalisation, the occurrence of invasive species and human pathogens experienced an exponential increase throughout the 20th century showing no signs of abating. Specifically, the rapid expansion of global trade and economic networks has significantly facilitated the extensive dispersion of invasive species and pathogens to distant locations from their original habitats via global networks [1]. This phenomenon has led to the establishment of self‐sustaining pathogen populations and their rapid propagation from the introduction sites, causing substantial impacts on the environment, economy and human welfare in the invaded regions. Moreover, human global economic activities have become a key driving force behind the transmission of human epidemics as well as the invasion and dissemination of alien species [2]. Actually, the movement of infected human hosts plays a crucial role in the spread of human pathogens beyond their original locations and the subsequent formation of invasions [3]. In addition to the human factor, it should be noted that these invasive species themselves can also function as carriers or hosts of human pathogens, thereby promoting large‐scale dissemination of human pathogens and epidemics. Hence, the rise in the likelihood of human infection by infectious pathogens and the expansion of their transmission range can be viewed as instances of biological invasion. In fact, there are striking similarities between the transmission and invasion processes of human pathogens and biological invasions in terms of basic concepts and processes [4].

The field of invasive biology investigates the impact of invasive species on the process of colonisation and transmission, emphasising the mechanisms of invasion, the range of diffusion and the influence of environmental factors on the invasion process [5]. Human epidemiology, on the other hand, is concerned with the occurrence, progression and distribution of infectious diseases in human populations, with a focus on the transmission mechanisms of pathogens between hosts, the pathogenicity of specific microbial strains, the identification of susceptible individuals and the role of environmental conditions in pathogen transmission [6].

However, the existing public health prevention and control system faces obvious ‘domain fragmentation’ when addressing pathogen transmission and biological invasion. In the field of infectious disease prevention and control, research mostly focuses on the single chain of ‘pathogen‐host‐transmission route’ (e.g., developing vaccines to block human‐to‐human transmission or implementing isolation to cut off contact transmission), but often overlooks that ‘the source spread of pathogens may be related to biological invasion’—for example, *Aedes albopictus*, the transmission vector of dengue virus, is a typical invasive species, and its global spread is closely linked to ‘unintentional carrying’ in international trade cargo; focusing only on virus control without blocking the mosquito’s invasion path makes it difficult to fundamentally curb recurrent epidemics. From the core principle of ‘prevention first’ in public health, current research has two key gaps: first, ‘insufficient risk prediction capacity’—existing models mostly predict the spread trend of infectious diseases or the invasion path of alien species independently, failing to integrate their associated mechanisms (e.g., being unable to use the spread model of invasive species to predict the potential transmission range of pathogens they carry in advance, making it hard for public health departments to formulate ‘proactive deployment’ prevention strategies); although the ecological field has rich survey data on biological invasion (e.g., distribution points of invasive species and spread speed), the two types of data have not been shared and integrated, failing to achieve a ‘1 + 1 > 2’ analysis effect.

By comparing the transmission and diffusion mechanisms of invasion biology and human epidemiology, it becomes evident that there is a certain degree of overlap in their research foci. Firstly, both disciplines follow a similar developmental stage in the study of invasion and transmission processes, employing common terminologies and mechanisms. Secondly, both disciplines recognise the resemblance between the transmission of pathogens in human populations and the transmission patterns of invasive species. Specifically, global trade and travel are identified as the primary means through which invasive species and pathogens are introduced, affecting vertebrate hosts and insect vectors. Consequently, many theoretical and empirical insights gained in invasion biology can be extended to the study of emerging pathogen epidemics. Similarly, invasive biology can also benefit from the knowledge gained through the study of emerging human infectious pathogens. Building upon these conclusions, this paper aims to explore the feasibility of further cross‐disciplinary collaboration between the two fields, focusing on the perspective of data modelling.

## 2 Mechanism Analysis

Invasion biology mainly adopts species distribution models (SDM) based on niche theory to predict the spatial distribution range of invasive species by integrating species survival and reproduction characteristics with environmental factors. Its mainstream analytical methods include the MaxEnt model, BIOCLIM model, generalised linear model (GLM) and random forest model [7]. In contrast, human epidemiology generally uses the susceptible‐infected‐recovered (SIR) model and the individual‐based Model (IBM) combined with spatial network structure theory to simulate the transmission process of infectious disease [8]. Although both disciplines focus on diffusion and distribution research, there are core differences in their diffusion models, rooted in the distinct differences in diffusion rates and lag times between infectious diseases and invasive species: infectious disease pathogens spread exponentially at a speed of 10^3^–10^4^ km per year (e.g., Zika virus, Ebola virus and West Nile virus) [9, 10], which is much higher than the diffusion rate of terrestrial invasive animals and plants (0.1–100 km per year) [11, 12]; meanwhile, invasive species usually have a long lag time, with decades elapsing between their introduction and rapid diffusion [13], whereas the lag time of dengue fever is relatively short and more observable. By dividing the life cycle stages of the pathogen, the lag time can be accurately defined and effective isolation and quarantine measures can be implemented.

Currently, cross‐disciplinary research between invasion biology and human epidemiology is limited by differences in diffusion characteristics, data availability and heterogeneity of information source. As a core tool in invasion biology for predicting susceptible habitats under climate change, species distribution models have limited applications in epidemiology, with only a few infectious disease pathogens (e.g., dengue fever, *Lassa virus*, *Mayaro virus*, *monkeypox virus* and *malaria parasites* such as *Plasmodium falciparum* and *Plasmodium vivax*) being used for transmission area analysis. The core reason for this limited application is the weak human‐to‐human transmission capacity of such pathogens: dengue fever is mainly transmitted through blood transfusion or vertical transmission; *Lassa virus* can only be transmitted through close contact with the body fluids of infected individuals; *Mayaro virus* is mainly transmitted through the bites of infected mosquitoes, and there is currently no conclusive evidence of its human‐to‐human transmission capacity and *monkeypox virus* can be transmitted through direct contact with infected individuals or respiratory droplets, but the scope of human‐to‐human transmission is usually limited [14]. The distribution of such pathogens is jointly determined by the ecological adaptability of all participating species in their transmission cycles and the unique diffusion limitations of each species, which also establishes a natural link between the habitat prediction methods of invasion biology and epidemiological research on vector‐borne diseases.

It is worth noting that the two disciplines are complementary in terms of data characteristics: invasion species research mainly relies on discovery location records, whereas epidemiological research has highly complete case tracking data, which can make up for the lack of follow‐up tracking data in invasion biology research. At present, only a few species (such as *Acacia saligna* and *zebra mussels*) in invasion species research have centralised and comprehensive datasets [15]. Therefore, combining invasion biology models with epidemiological data has become a potentially important direction for exploring the diffusion rules of vector‐borne diseases.

Although the theoretical and methodological links between invasion biology and human epidemiology have been initially recognised, there remains a key research gap: in existing studies, few have combined SDM‐based vector suitable habitat prediction with spatial econometric models to quantitatively analyse the direct and indirect spatial effects of vector habitats on the prevalence of vector‐borne diseases. Specifically, there is a lack of relevant empirical research that uses the MaxEnt model to construct mosquito threat indices based on the distribution of high‐suitability habitats, and further applies the spatial Durbin model (SDM) to decompose the local and cross‐regional spillover effects of mosquito suitable habitats on dengue fever cases. To fill this research gap, this study takes dengue fever, a typical vector‐borne disease transmitted by *Aedes aegypti* and *A. albopictus*, as the research object, and sets two core research objectives: (1) Based on the existing distribution records of the two *Aedes* species and environmental factor variables, use the MaxEnt model to predict their suitable habitat distribution in China, construct city‐scale *Aedes* threat indices (proportion of high‐suitability habitat area) and clarify the spatial distribution characteristics and coexistence patterns of the two *Aedes* species; (2) combine the spatial distribution data of dengue fever cases in China, apply SDM to verify the spatial correlation between *Aedes* suitable habitats and dengue fever epidemic areas, quantitatively analyse the direct effects (impacts of local *Aedes* high‐suitability habitats on local dengue fever cases) and indirect effects (cross‐regional spatial spillover effects of *Aedes* habitats in adjacent regions on local dengue fever cases) of the threat indices of the two *Aedes* species on dengue fever prevalence, and further reveal the driving mechanism of *Aedes* habitat distribution on the spatial epidemic pattern of dengue fever.

Building on these objectives, the main contributions of this study are threefold: (i) a coupled MaxEnt–spatial Durbin model framework that converts gridded habitat‐suitability outputs into city‐scale Aedes threat indices, enabling a quantitative decomposition of the direct and indirect (spillover) effects of Aedes habitat suitability on dengue incidence in China; (ii) a dual weight‐matrix design, based on geographic distance and transportation networks and diagnosed with global Moran’s *I*, that distinguishes the local transmission channel from the cross‐regional dissemination channel of the two Aedes vectors and (iii) a forward‐looking scenario analysis that reprojects the calibrated MaxEnt model under three SSP pathways (SSP1‐2.6, SSP2‐4.5 and SSP5‐8.5) for the mid‐century (2041–2060) and end‐of‐century (2081–2100) periods, and quantifies the prospective shifts in habitat suitability and spillover dynamics of both species.

## 3 Methods

### 3.1 Selection of Epidemic Pathogens

Over the past five decades, the global incidence of dengue fever has soared by 30 times, presenting a significant public health challenge. With an estimated 50 million to 100 million infections occurring annually in over 100 endemic countries, nearly half of the world’s population is at risk. There is currently no specific antiviral treatment for dengue fever, but early detection and access to appropriate medical care can reduce mortality rates to below 1%. Therefore, it is crucial to effectively identify the geographical hotspots of *A. aegypti* and *A. albopictus* aggregation and implement preventative measures to combat dengue fever, mitigating the health risks posed by the dengue virus.

Dengue fever is caused by the dengue virus, transmitted primarily by *A. aegypti* and secondarily by *A. albopictus*. Climate is key to its large‐scale prevalence: the 20°C–30°C range optimises *A. aegypti* growth across all life stages (egg, larva, pupa, adult); rainfall‐formed stagnant water directly boosts mosquito density by providing breeding sites and factors, such as humidity and air pressure, indirectly affect transmission by regulating mosquito adaptability, biting behaviour and adult survival.

Ecologically, *A. aegypti* is highly domestic, preferring indoor residential areas—especially near dark humid breeding environments. Its larvae mainly breed in household water storage containers (metal barrels: 24.22% and water tanks: 23.34%). This close association with human settlements makes *A. aegypti* density and virus‐carrying rate core indicators of regional dengue prevalence intensity.

For diffusion, *A. aegypti* (origin: Africa) and *A. albopictus* (origin: Asia) have spread globally over decades via transportation (airplanes and ships) and mosquito egg‐carrying commodities (e.g., tires). Meanwhile, commerce and tourism drive cross‐regional movement of infected people, expanding the infection source. Urbanisation‐induced land use changes (e.g., converting farmland/mountains to construction land) also alter *Aedes* habitats and breeding environments, reshaping dengue transmission patterns.

*Aedes*, the largest genus in the Culicidae family, comprises over 40 subgenera and more than 1000 species. Among these, *A. albopictus* and *A. aegypti* are the most prevalent species known to transmit a range of infectious diseases, including yellow fever, dengue fever, chikungunya fever, Zika virus disease and Rift Valley fever. Although *A. aegypti* poses a greater threat in the transmission of dengue fever, *A. albopictus* has established a broad geographic presence as an invasive species due to its biological versatility and adaptability. The global expansion of *A. albopictus* is primarily driven by human activities, such as intensified intercontinental trade, facilitating its dispersal. Given its increasing prevalence in diverse environments, together with its capacity to harbour various arboviruses, it is essential to evaluate its potential in spreading highly contagious pathogens.

The transmission of the dengue virus rarely occurs through human‐to‐human contact, emphasising the critical role of *A. aegypti* and *A. albopictus* as primary vectors. The distribution of the dengue virus aligns with the spread pattern typical of invasive species, underscoring the relevance of species distribution models in assessing suitable environments for their dissemination. Furthermore, the availability of comprehensive tracking data on dengue virus transmission enables the application of spatial models to forecast the occurrence of dengue fever based on estimations of suitable habitats. This study aims to delve deeper into whether the prediction of dengue fever incidence can be enhanced by integrating spatial models with assessments of environmental suitability.

### 3.2 Model and Data

In this study, the estimation of the species distribution of two *Aedes species* was conducted using the MaxEnt method. MaxEnt was chosen due to its ability to generate robust models even with limited sample sizes [16]. A critical advantage when early epidemic data are scarce, unlike BIOCLIM, which requires large, balanced presence‐absence datasets to avoid bias; second, as a habitat‐based model, it uses known species and environmental data and transcends administrative divisions. Notably, pathogen hosts (e.g., mosquitoes) in epidemiology typically have ‘known presence but unknown absence’ data, only epidemic site records are available, and non‐epidemic surveys for negative data are unfeasible. This is where MaxEnt outperforms presence‐absence models (e.g., GLM): it independently constructs a complete prediction framework without missing data, whereas GLMs fail to generate reliable results when negative data are unavailable, making MaxEnt uniquely suited to this epidemiological data constraint.

For analysing the spatial diffusion impact of *Aedes* mosquitoes, the Spatial Durbin Model (SDM) was utilised. The SDM is a spatial econometric model that takes into account spatial interaction and is widely applied in spatial analyses of various socio‐economic phenomena. By incorporating the spatial lag effect into the traditional linear regression model, the SDM captures the spatial dependence of explanatory variables. In the SDM, two fundamental concepts, namely, direct effect and indirect effect, represent different mechanisms of spatial action:

Direct Effects: Direct effects pertain to the direct impact of explanatory variables within a model on the explained variables within the same geographical area. Essentially, it represents the direct influence that a change in an explanatory variable has on the interpreted variable in that specific area, without taking into account the influence of other areas. The direct effect highlights the independent role of explanatory variables, disregarding spatial interactions.

Indirect Effects: Indirect effects, also referred to as spatial spillover effects, describe the influence of explanatory variables on the dependent variable through neighbouring regions. This effect captures spatial interactions, illustrating how changes in explanatory variables in one region affect other regions through spatial connections, ultimately impacting the dependent variable. By unveiling the interdependence and influence between regions, indirect effects shed light on the positive or negative externalities present in spatial relationships.

By analysing both direct and indirect effects, we can gain a deeper understanding of the impact of *Aedes* mosquitoes on the spatial spread of dengue fever.

#### 3.2.1 Data Collection

As the core tool for this habitat prediction, the MaxEnt model relies on two categories of key input data: *Aedes* species distribution data and environmental and human activity variables. The sources, processing and roles of these datasets are detailed below:

##### 3.2.1.1 *Aedes* Species Distribution Data

Distribution data for the two target *Aedes* species were sourced from the Global Biodiversity Information Facility (GBIF) database, which can be accessed at https://www.gbif.org/occurrence/search?q=Aedes%20species. Using ArcGIS, we performed preprocessing by filtering duplicate, ambiguous (e.g., incomplete coordinates) and geographically adjacent records to reduce sample variance, with 81,303 occurrence records retained for *A. aegypti* and 141,104 for *A. albopictus* after the filtering process. The refined data were standardised to a 2.5 arc‐minute resolution (approximately 5 km × 5 km grid) and saved in CSV format for model construction.

##### 3.2.1.2 Environmental and Human Activity Variables (For Aedes Species Habitat Suitability Prediction)

###### 3.2.1.2.1 Environmental Impact Variable

Data collected from the World Climate Organization regarding current climate conditions, including key variables, such as temperature and precipitation patterns, were utilised to forecast the future aggregation of *Aedes* mosquitoes, which can be accessed at https://www.worldclim.org/data/index.html. Nineteen environmental variables were considered, with all layers resampled to a resolution of 2.5 arc minutes (approximately 5 km × 5 km) for the Chinese mainland. This was achieved through bilinear interpolation and clipping techniques.

###### 3.2.1.2.2 Land Use Data

Recognising that *A. aegypti* mosquitoes typically breed in close proximity to human habitation, land use data (LUCC data) with a 30 m spatial resolution was integrated into the environmental impact variables, which can be accessed at https://www.resdc.cn/DOI/DOI.aspx?DOIid=54. The LUCC data were obtained from the Resource and Environment Data Center of China Academy of Sciences, derived from LUCC data in 2018 and updated using remote sensing imagery from the Gaofen No. 1 satellite between 1 July and 1 August 2020. The land use data categorise areas into cultivated land, woodland, grassland, water bodies, constructed areas and unused land, with the aim of distinguishing human activity zones from natural environments.

###### 3.2.1.2.3 Human Activity Data

This dataset was leveraged to incorporate social and economic indicators of human activities influencing the spread of dengue virus. It comprises the kilometre grid datasets showing the spatial distribution of China's GDP (https://www.resdc.cn/data.aspx?dataid=252). The data were instrumental in constructing the spatial correlation matrix within the spatial model, employing geographical and traffic distances between cities to establish the spatial weight matrix. The weight matrix takes into account the spread routes of *Aedes* mosquitoes, including both aerial distances (flight distances) and man‐made pathways.

#### 3.2.2 Species Distribution Modelling

For the setup of the MaxEnt model, this study adopted the model's default cross‐validation strategy with specific parameter settings to meet the review requirements for model operation. Specifically, the distribution data of *A. aegypti* and *A. albopictus* were each partitioned into a training set (for model construction) and a test set (for model validation) at a proportional ratio of 90% for training and 10% for testing. Moreover, a 10‐fold cross‐validation method was implemented for model validation in MaxEnt, the maximum number of iterations was set to 100,000 and all remaining parameters (including the regularisation multiplier) were kept as default. This partitioning and parameter setting approach ensures the objectivity and reliability of the validation results. Additionally, the area under the receiver operating characteristic curve (AUC) was used to evaluate the predictive accuracy of the model.

#### 3.2.3 Spatial Econometric Analysis

In the spatial econometric analysis framework of this study, the dengue dataset and *Aedes* threat indices serve as the core dependent variable and independent variables, respectively.

##### 3.2.3.1 Dependent Variable: Dengue Dataset

The data source is the Chinese Disease Prevention and Control Information System (NNDRS) from 2005 to 2020. The included cases consist of laboratory‐confirmed cases and clinically diagnosed cases, excluding Hong Kong, China, Macao, China and Taiwan, China. Population data are from the National Bureau of Statistics of China. It was utilised to define the dependent variable dengue within the spatial model and determine the proportion of total dengue cases relative to the population in each city. In the past 5 years, reported cases were mainly from Guangdong Province and Yunnan Province. In 2019, Guangdong Province began to report more than 50 cases starting from EW23 and continued until EW47, with a cumulative total of 5574 cases reported. The highest peak was at EW42, with 514 cases reported.

##### 3.2.3.2 Independent Variables: *Aedes* Threat Indices

###### 3.2.3.2.1 Precondition for Index Calculation: Prediction of *Aedes* Suitable Habitats

The suitable habitat for two *Aedes* species: The training set and the test set were randomly divided multiple times to obtain a prediction model with a higher AUC value for both sets. This model was used to identify the suitable habitat for the mosquitoes. The suitable habitat was determined based on the probability of survival, with the high‐suitability habitat having a survival probability greater than 0.6, the medium‐suitability habitat between 0.3 and 0.6 and the low‐suitability habitat below 0.3.

###### 3.2.3.2.2 Specific Definitions of the Two Threat Indices

The threat index of *Aedes* mosquitoes in different cities: This index was used as an explanatory variable to determine if a city falls within the suitable habitat for *Aedes* mosquitoes. The objective of this study was to investigate the spatial relationship between the suitable habitat for *Aedes* mosquitoes and the infection rate of dengue fever in cities. Additionally, it aimed to analyse the direct and indirect effects of this relationship. The Aeg threat index measured the area occupied by *A. aegypti* in the city’s high‐suitability habitat relative to the city’s total area. Similarly, the Alb threat index calculated the area occupied by *A. albopictus* in the city’s high‐suitability habitat using the MaxEnt result also relative to the city’s total area.

#### 3.2.4 SSP Scenario Data and Future Projections

To project the spatial trajectory of Aedes suitable habitats and dengue risk under future climate and socioeconomic changes, future environmental and socioeconomic datasets under three shared socioeconomic pathways (SSP1‐2.6, SSP2‐4.5 and SSP5‐8.5) for the mid‐century period (2041–2060) and the end‐of‐century period (2081–2100) were integrated into the modelling framework.

##### 3.2.4.1 Future Data Collection and Preprocessing

Climatic Variables: Multimodel ensemble projections of bioclimatic variables (Bio1–Bio19) derived from CMIP6 GCMs (EC‐Earth3‐Veg, GFDL‐ESM4 and MRI‐ESM2‐0) were obtained from WorldClim v2.1 at a spatial resolution of 2.5 arc‐minutes.

Land Use and Socioeconomic Grids: Spatially explicit future land‐use datasets (LUH2) were processed to extract future construction land (lucc_05) and woodland (lucc_02) layers. Gridded population and GDP projections under corresponding SSP scenarios were retrieved from RESDC and GHSL to update future city‐level socioeconomic centres and traffic/economic spatial weight matrices (*W*_t_).

##### 3.2.4.2 Multi‐Scenario Predictive Coupling

Habitat Suitability Projection: The calibrated MaxEnt model was executed on the future climate‐LUCC gridded layers under each scenario (SSP1‐2.6, SSP2‐4.5 and SSP5‐8.5) for the mid‐century (2041–2060) and end‐of‐century (2081–2100) periods. Updated city‐scale threat indices (${\text{aeg}}_{i,t}^{\text{SSP}}$ and ${\text{alb}}_{i,t}^{\text{SSP}}$) were recalculated as the predicted proportion of high‐suitability habitat area within each municipality.

Spatial Risk Simulation: Incorporating the prospective threat indices and updated spatial weight matrices, the spatial Durbin model (SDM) was applied to project prospective direct effects (local transmission risk) and indirect spillover effects (cross‐regional dissemination risk) across Chinese cities.

### 3.3 Selection of Variables Required for Suitable Habitat

Due to the high collinearity among 19 environmental variables, the YANG method was employed in this study to address this issue. Initially, MaxEnt was utilised to assess the significance of different variables, and those with a contribution of less than 1% were excluded. Subsequently, a correlation test was conducted on the remaining variables. In cases where two variables exhibited a correlation coefficient exceeding 0.8, the one with the lower contribution was eliminated to facilitate dimensionality reduction. Finally, the retained variables were integrated with land use data, and the environmental variables utilised by the two *Aedes* species are presented in Table 1.

**TABLE 1:** Environmental variables used by the two *Aedes* species.

Species	Environmental factor abbreviation	Environmental factor name	Contribution degree
*Aedes aegypti*	bio_3	Isothermal property	39.6
	lucc_05	Land for construction	38.2
	bio_16	Precipitation in the wettest quarter	6.6
	bio_11	Average temperature of the coldest quarter	3.4
	bio_6	The lowest temperature in the coldest month	2.4
	bio_7	Annual average temperature variation range	2.2
	bio_4	Standard deviation of seasonal temperature changes	1.7
	bio_14	Precipitation in the driest month	1.7
	bio_2	Monthly average temperature difference between day and night	1.5
*Aedes albopictus*	bio_12	Annual precipitation	69.2
	bio_11	Average temperature of the coldest quarter	13.2
	bio_10	The average temperature of the warmest quarter	3.3
	bio_3	Isothermal property	3.3
	bio_1	Annual average temperature	2.4
	lucc_05	Land for construction	2
	lucc_02	Woodland	1.8
	bio_19	Precipitation in the driest month	1.2

Through an analysis of the contributions of diverse environmental variables, distinctions in the active regions of the two *Aedes* species were discerned. *A. aegypti* not only displayed a high affinity for temperature but also demonstrated a significant reliance on construction land, indicating a preference for human habitation and reliance on humans as a primary blood source. On the other hand, *A. albopictus* predominantly focused on annual precipitation, implying its presence within a broader suitable range as long as the annual precipitation aligns with its requirements. This examination aided in comprehending the distribution and behavioural patterns of the two *Aedes* mosquito species across varying environmental conditions.

## 4 Results

### 4.1 Suitable Habitat Analysis

Based on the distribution data of *A. aegypti* and *A. albopictus* as well as relevant environmental factors, this study employed the MaxEnt model to predict their potential suitable habitats. The AUC value evaluation of the model’s prediction results is shown in Figure 1, with the optimal AUC value for *A. aegypti* being 0.886 and that for *A. albopictus* being 0.891. Both values are significantly greater than 0.8, indicating that the model’s predictions of the potential suitable habitats for the two *Aedes* species are highly reliable.

**FIGURE 1:**
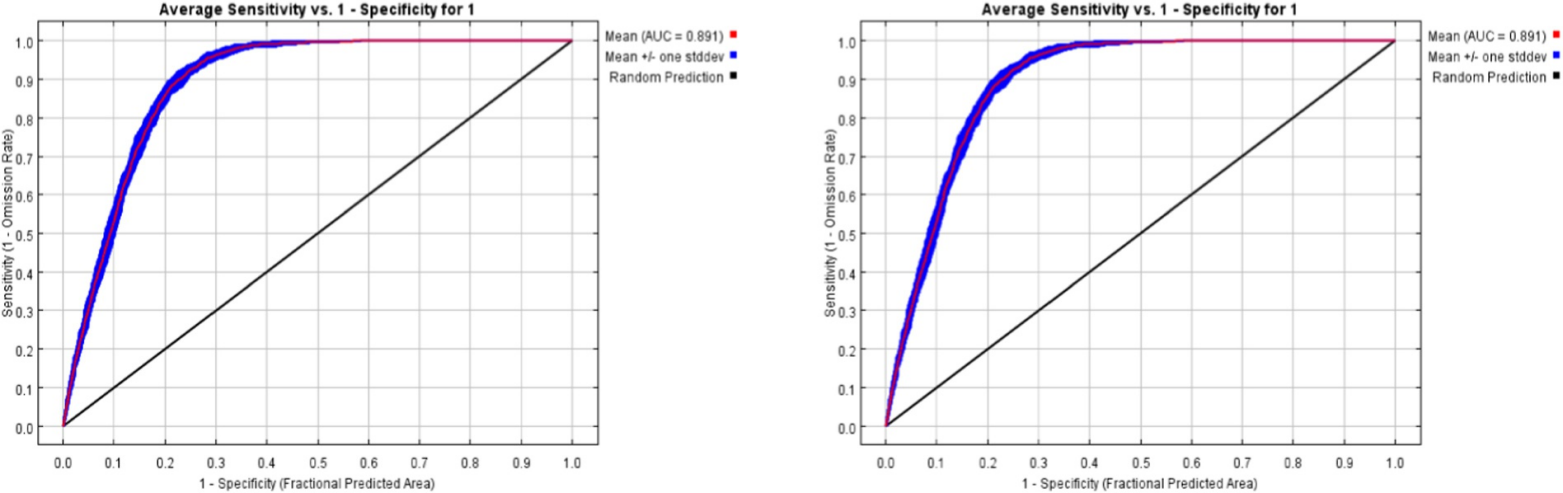
ROC curves of model predictions for the two *Aedes* species. *Aedes aegypti* (left) and *Aedes albopictus* (right).

Based on the current resolution of 2.5 arc minutes (approximately 5 km × 5 km), the survival probability and the pixel area product were utilised to calculate the threat indicators for both types of *Aedes* mosquitoes in each city.

*Aedes aegypti* predominantly inhabits regions such as Hainan Province, western and southern Yunnan Province and southeastern Guangdong Province. These areas experience a subtropical monsoon climate characterised by warmth and humidity, which aligns with its optimal growth temperature requirement of 20°C–30°C and creates ideal environmental conditions for its survival. Additionally, the frequent rainfall in these regions easily forms stagnant water, providing ideal breeding sites for its aquatic developmental stages (egg, larva and pupa) and further supporting the persistence of its population. On the other hand, the distribution range of *A. albopictus* extends beyond that of *A. aegypti*. Apart from subtropical monsoon climate regions, such as Yunnan, Hainan, Guangdong and Fujian, *A. albopictus* can also be found in Guizhou and Sichuan in the southwest and Hunan and Jiangxi in the central and southern provinces as well as Shaanxi, Shanxi, Beijing and Liaoning in the north. This broader distribution is associated with its stronger environmental adaptability—even in parts of northern regions where temperatures are too low or diurnal temperature variations are significant, its eggs can withstand drought for several months. Once temperature and rainfall conditions become favourable, the eggs quickly hatch and develop, further supporting their ability to thrive across a wider geographic range. Figure 2 exclusively depicts the projected distribution of both *Aedes* mosquito species in China’s high‐suitability habitats.

**FIGURE 2:**
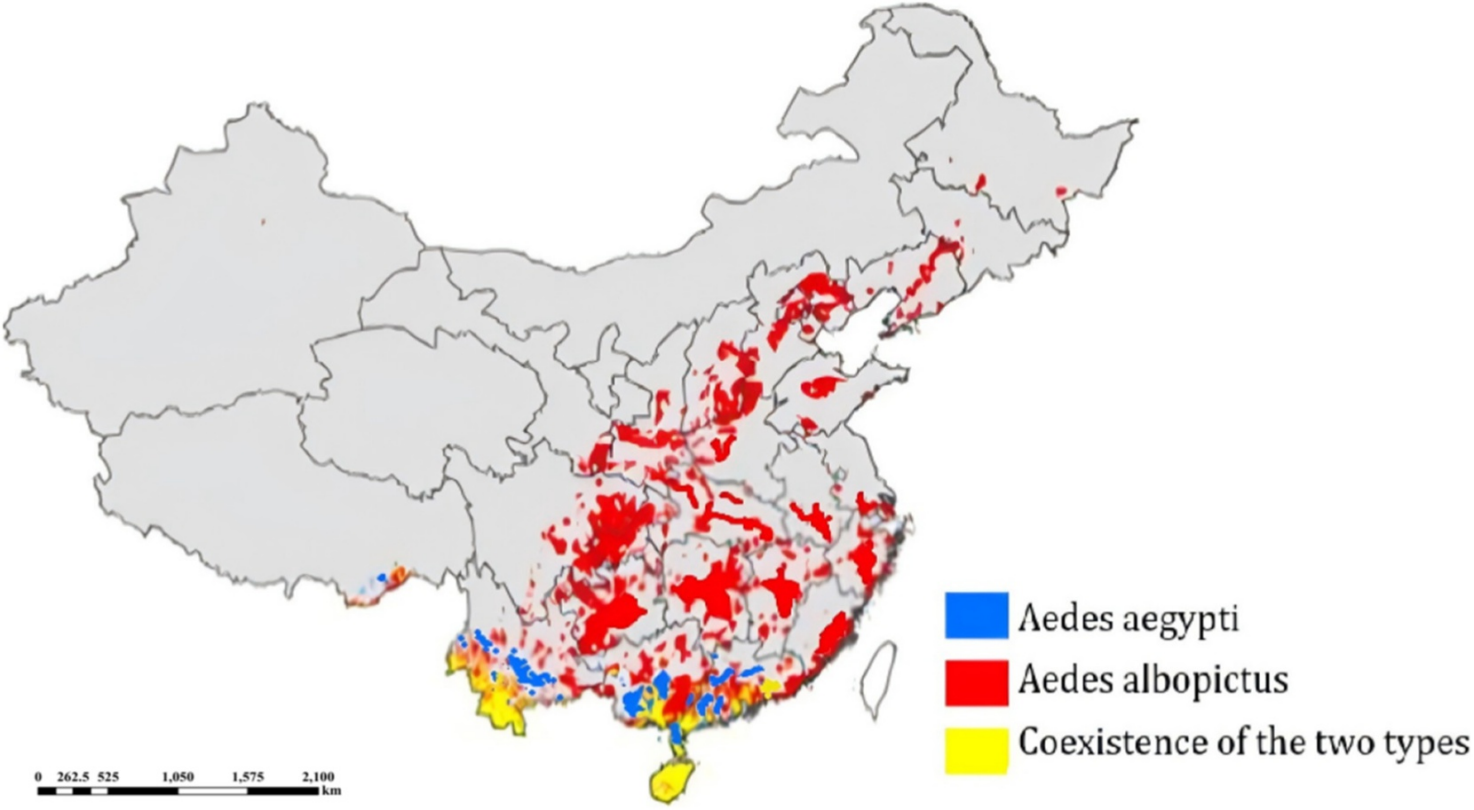
Predicted distribution of high‐suitability habitats of *Aedes aegypti* and *Aedes albopictus* in China (based on the MaxEnt model). This figure shows the predicted spatial distribution of high‐suitability habitats of *A. aegypti* and *A. albopictus* based on the MaxEnt model, not real‐time surveillance data of mosquito populations. The model uses environmental variables (Table 1) and occurrence records from the GBIF database to generate predictions.

### 4.2 The Spatial Correlation Between *Aedes* Distribution and Dengue Fever Outbreak Regions

Through the application of the biological distribution model in invasion biology, the study calculated the suitable habitats for *A. aegypti* and *A. albopictus* and derived threat indicators for these two species across various cities and regions based on their respective suitable habitats. The analysis also integrated data on dengue fever in China, providing further insights into the impact of *A. aegypti* and *A. albopictus* on the transmission and prevalence of the disease. Notably, the influence of *Aedes* mosquitoes extends beyond their immediate habitats as they have the potential to spread through three primary modes of transmission: (1) Airborne transmission, as *Aedes* mosquitoes are capable of flying under favourable climatic conditions, carrying the virus to new areas over short distances; (2) Waterborne dispersal, as *Aedes* mosquitoes commonly lay their eggs in water bodies, such as puddles, vases and tires, with the resulting larvae and adults dispersing short distances through water bodies, underscoring the critical role of water in the mosquitoes’ life cycle; (3) anthropogenic transmission, emphasising the significant impact of human activities on the spread of *Aedes* mosquitoes. Eggs, larvae or adult mosquitoes can be transported to new areas through trade and travel, particularly in the context of international commerce and tourism, leading to widespread dispersion of *Aedes* mosquitoes.

Hence, the prevalence of dengue fever could potentially exhibit spatial diffusion dynamics. To ascertain the spatial interplay between the habitats conducive to *Aedes* mosquitoes and dengue fever outbreak zones, along with discerning the divergent diffusion patterns of the two *Aedes* mosquito species, a spatial econometric model was employed to validate the reliability of the aforementioned findings. Furthermore, the spatial dependence model (SDM) was employed for verification. Subsequently, the aggregate impact was disentangled into direct and indirect effects to scrutinise the viral transmission capacity of *Aedes* mosquitoes within their suitable habitats as well as their transmission potential through diffusion pathways.

### 4.3 Spatial Distribution Characteristics of Dengue Fever Incidence Areas

Based on the findings by Yu‐Juan et al. [17], this study examined the spatial distribution characteristics of indigenous and imported dengue fever cases in mainland China spanning the period from 2005 to 2020 through the application of ArcGIS 10.2 software (as illustrated in Figure 3). From 2005 to 2020, all provinces in China, except for Tibet and the regions of Hong Kong, China, Macao, China and Taiwan, China, reported cases. These cases involved 285 cities and 1417 counties, showing significant regional clustering. The five provinces with the highest number of reported cases were Yunnan with 20,740 cases, Guangdong with 10,320 cases, Guangxi with 2166 cases, Fujian with 1835 cases and Chongqing with 1536 cases, accounting for 87.75% of the total number of cases. The results depicted in Figure 3 reveal that indigenous occurrences of dengue fever within China are predominantly concentrated in Guangdong and Yunnan provinces. According to pertinent data, the incidence of dengue fever in these two provinces represented 74.01% and 13.74% of the national total, respectively. Subsequently, the provinces with higher case numbers included Guangxi, Fujian, Zhejiang, Chongqing and Jiangxi. The collective indigenous dengue fever cases across these seven provinces constituted merely 1.3% of the aggregate. Noteworthy is the dengue fever outbreak that transpired in Jining City, Shandong Province, in 2017, marking the northernmost documented manifestation of dengue fever in China. Furthermore, in 2019, indigenous occurrences of dengue fever were widespread across 266 counties (districts) in 74 cities within 13 provinces, autonomous regions and centrally administered municipalities.

**FIGURE 3:**
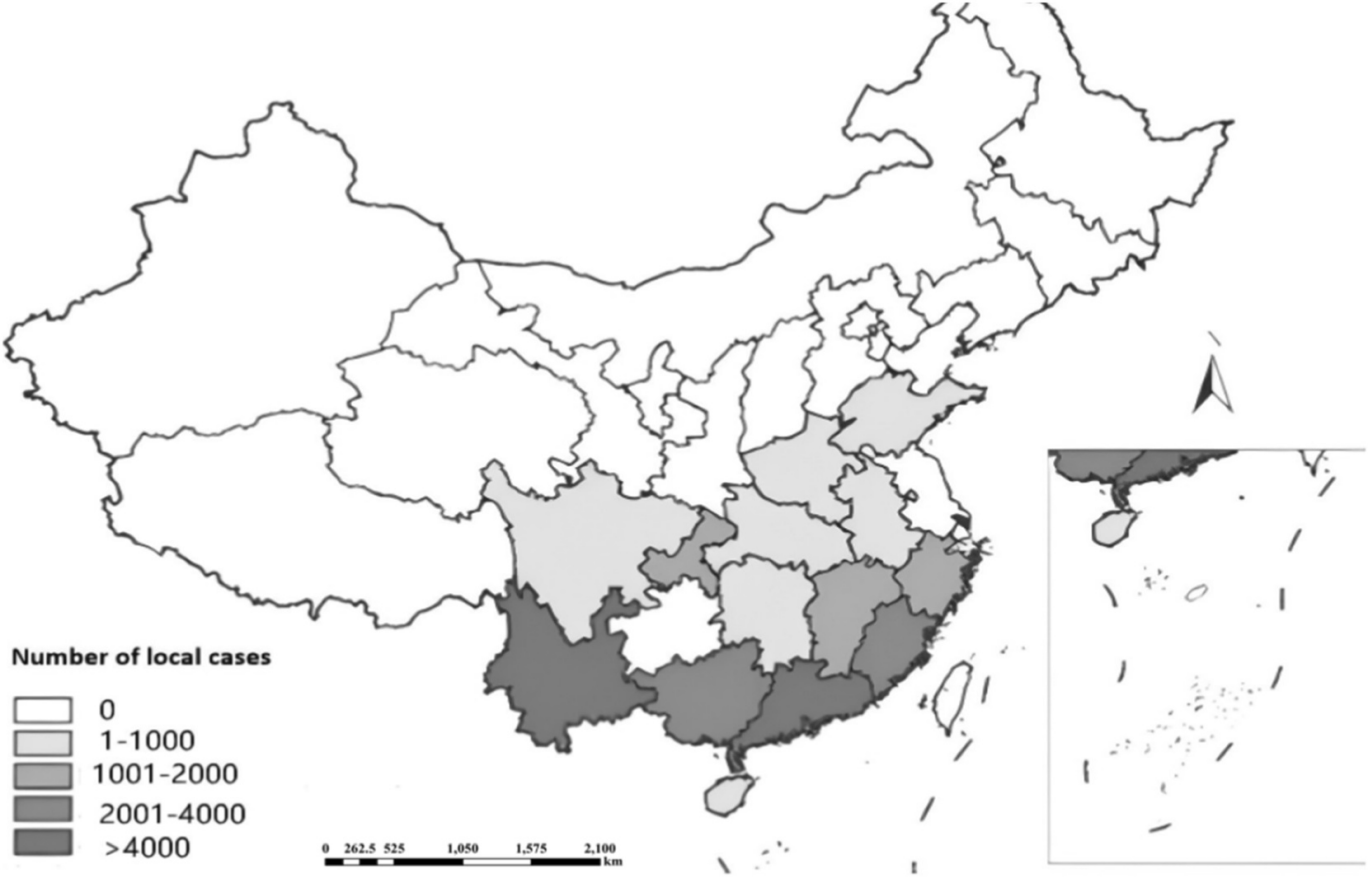
Dengue fever incidence area.

Through a comparison of the distribution of *Aedes* mosquitoes depicted in Figure 2 with the diagram illustrating the outbreak areas of dengue fever in Figure 3, it becomes evident that the regions where *Aedes* mosquitoes aggregate are highly correlated with the areas experiencing dengue fever outbreaks. In China, the provinces with the highest number of reported dengue fever cases are predominantly Guangdong and Yunnan Provinces—these regions also coincide with the most densely populated habitats of *A. aegypti* and *A. albopictus*.

From a climatic perspective, South China (including Guangdong, Guangxi and Hainan), characterised by tropical and subtropical climates, has seen its annual average temperature rise by 0.55°C since 1997 compared to the previous 30 years, with the number of days suitable for dengue fever transmission reaching an average of 210 days per year. This climatic feature is highly compatible with the biological needs of *Aedes* mosquitoes: For of *A. aegypti*, the optimal temperature range of 20°C–30°C and stable diurnal temperature variation accelerate larval development and increase the biting frequency of adult mosquitoes; although *A. albopictus* exhibits stronger temperature adaptability, the abundant precipitation in this region provides natural stagnant water (e.g., in tree holes and bamboo tubes) that further boosts its population density, creating favourable conditions for dengue transmission characterised by ‘high temperature + suitable temperature variation + sufficient breeding sites’.

Epidemiological studies confirm that dengue fever is an acute mosquito‐borne infectious disease caused by the dengue virus (DENV), transmitted primarily by *A. aegypti* and *A. albopictus*. Its transmission efficiency relies on the spatial coupling of ‘*Aedes* density—climate suitability—virus input volume.’ Consequently, as the distribution range of these two *Aedes* species continues to expand in China (e.g., *A. albopictus* spreading northward to Shaanxi and Liaoning Provinces), coupled with climate warming extending the period suitable for transmission, the dengue virus will gain broader transmission vectors and a longer transmission window, leading to a sustained increase in the risk of further dengue fever spread.

The spatial Durbin model, an econometric model that incorporates spatial dependence, is utilised to analyse the diffusion effect within geographical areas. Initially, we examine whether there is spatial clustering and diffusion of the dengue virus. Given the limited likelihood of dengue virus transmission from person to person, any observed spatial aggregation and diffusion would more plausibly be attributed to the spatial distribution and diffusion patterns of *Aedes* mosquitoes. This serves as a foundation for subsequent analyses of the dissemination of dengue fever through areas suitable for *Aedes* mosquitoes.

In order to quantify and test the spatial correlation, we employed the Moran index. The Moran index, also known as Moran's *I*, is a statistical indicator for measuring spatial autocorrelation within spatial datasets. It is frequently employed to assess the similarity between neighbouring regions in terms of spatial patterns. The calculation formula for the Moran index is as follows:

$$I=\frac{N}{W}\frac{\sum_{i=1}^{N}\sum_{j=1}^{N}w_{ij}\left({\text{Dengue}}_{i}-\bar{\text{Dengue}}\right)\left({\text{Dengue}}_{j}-\bar{\text{Dengue}}\right)}{\sum_{i=1}^{N}{\left({\text{Dengue}}_{i}-\bar{\text{Dengue}}\right)}^{2}},$$

where *N* represents the number of geographical units, the double summation of the spatial weights signifies the summation of all elements within the weight matrix, *w*_*ij*_ denotes the spatial weight between geographical units *i* and *j* and Dengue_*i*_ and Dengue_*j*_ are the values of dengue incidence in cities *i* and *j*. The numerator segment of the formula computes the product of the discrepancy between each pair of neighbouring regions, whereas the denominator segment encompasses the population variance of the observed value. The Moran’s *I* statistic ranges between −1 and 1, with positive values indicating positive spatial autocorrelation, negative values representing negative spatial autocorrelation and values close to zero signifying spatial randomness.

The findings demonstrate that within the framework of two distinct spatial weight matrices, the global Moran index utilising geographical distance as the spatial weight matrix stands at 0.305 (Figure 4), whereas the Moran’s *I* index utilising traffic distance as the spatial weight matrix is 0.206. Both outcomes strongly reject the null hypothesis of no spatial correlation at a significance level of 0.1%. Consequently, it is plausible to infer that there exists a spatial diffusion effect in the occurrence of dengue cases.

**FIGURE 4:**
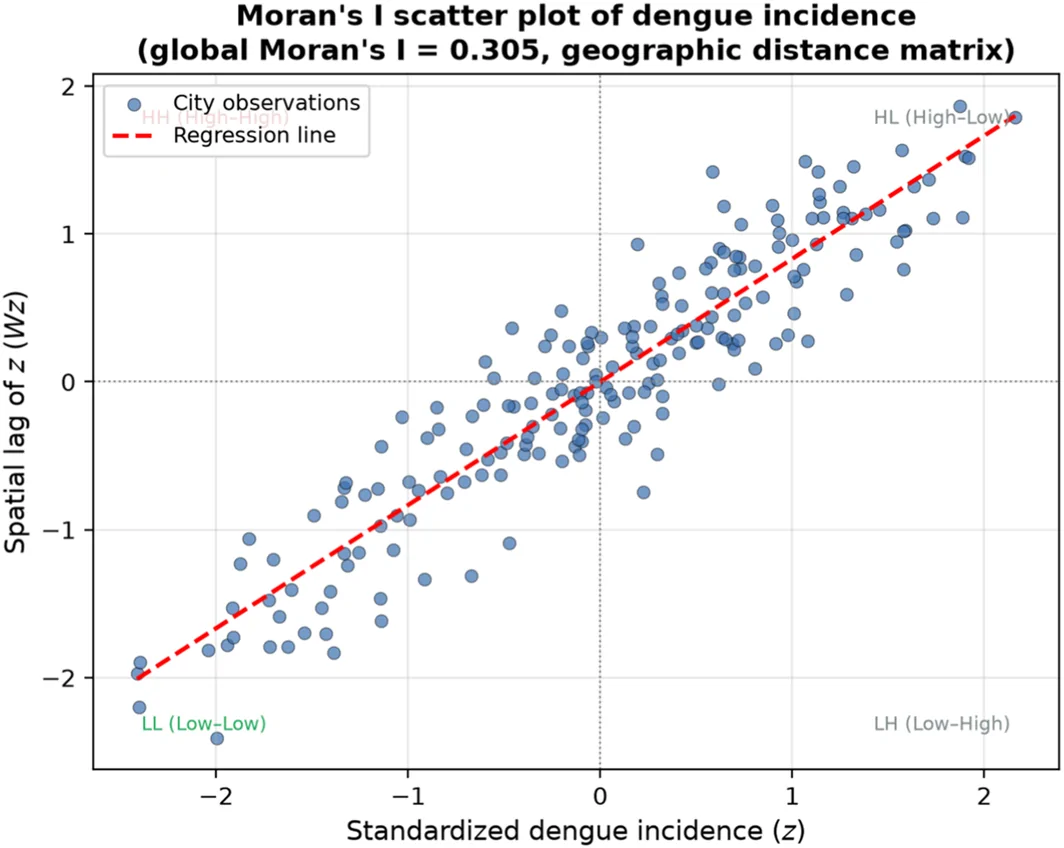
Moran's *I* scatter plot of dengue incidence across Chinese cities (global Moran’s *I* = 0.305 under the geographic distance matrix).

### 4.4 Spatial Durbin Model Analysis

In the spatial Durbin model (SDM), the spatial lag term—typically an intermediary variable quantifying ‘the aggregated risk status of neighbouring geographic areas’, such as average *Aedes* density or dengue incidence near the target area in epidemiological studies—has a coefficient that serves as an indicator of the indirect effect, measuring how strongly neighbouring risks spill over to the target area. This indirect effect elucidates the spatial interdependency of infectious diseases by explaining cross‐region risk propagation: for example, *Aedes* mosquitoes migrating from rain‐favoured neighbouring areas (via wind or human transport) or dengue patients travelling from epidemic regions can trigger local transmission in the target area, with SDM quantifying this spread to distinguish it from direct effects (e.g., local *Aedes* density impacting local cases). Particularly in vector‐borne disease studies (e.g., dengue and malaria), this indirect effect is often conceptualised as a disease diffusion mechanism—for example, simulating how a dengue outbreak in one city spreads to a neighbouring one via vectors or human mobility—helping researchers and practitioners understand how key epidemiological variables geographically disseminate via the spatial lag term to influence dependent variables such as local dengue incidence.

The rationale underpinning the utility of the spatial Durbin model resides in its capacity to partition the total effect of variables into direct and indirect components, with the latter effectively embodying a diffusion mechanism. Consequently, the spatial Durbin model emerges as a suitable choice for scrutinising both the localised direct impact of *Aedes* mosquitoes on dengue cases and the diffusive influence exerted by *Aedes* mosquitoes originating from other localities. The formulation of the specific model is outlined as follows:

$${\text{Dengue}}_{i}=\rho \sum_{j=1}^{N}w_{ij}{\text{Dengue}}_{j}+\varphi {\text{aeg}}_{i}+ϴ\sum_{j=1}^{N}w_{ij}{\text{aeg}}_{j}+\alpha {\text{alb}}_{i}+\beta \sum_{j=1}^{N}w_{ij}{\text{alb}}_{j}+\varepsilon_{i},$$

where dengue represents the total incidence of dengue fever in the city, *w*_*ij*_ is the spatial weight matrix constructed before and aeg and alb are the threat indices of *A. aegypti* and *A. albopictus*, respectively, each defined as the proportion of the city’s high‐suitability habitat area relative to the city’s total area (as shown in Table 2).

**TABLE 2:** Decomposition of the effects of two *Aedes* species on dengue fever cases: Direct, indirect and total effects.

Explanatory variables	Spatial weight matrix *W* = *W*_*d*_			Spatial weight matrix *W* = *W*_*t*_		
	Direct effects	Indirect effects	Total effects	Direct effects	Indirect effects	Total effects
Aeg	0.0755***	0.0027**	0.0782***	0.0722**	0.0015	0.0737*
	(3.157)	(1.953)	(3.834)	(1.972)	(1.310)	(1.712)
Alb	0.0134*	0.0095**	0.0229*	0.0125*	0.0087*	0.0212**
	(1.673)	(2.198)	(1.877)	(1.734)	(1.832)	(2.117)

*Note:* Unless otherwise specified, the values in parentheses represent the *t*‐statistic of the corresponding variable.

*, **, *** represent significance levels of 10%, 5%, and 1%, respectively.

As indicated by the analysis of Table 2 and Figure 5, *A. aegypti* exhibits a direct effect of 0.0755 (*p* < 0.01) on local dengue transmission. This implies that a 10% increase in the proportion of high‐suitability habitats for *A. aegypti* in a city would lead to a significant rise of 0.755% in the local dengue incidence rate. Its indirect effect (i.e., the impact of *A. aegypti* in neighbouring regions on local dengue) is 0.0027 (*p* < 0.05), further confirming that *A. aegypti* is a core driver of local dengue outbreaks, even a slight increase in its density may trigger a notable growth in dengue cases.

**FIGURE 5:**
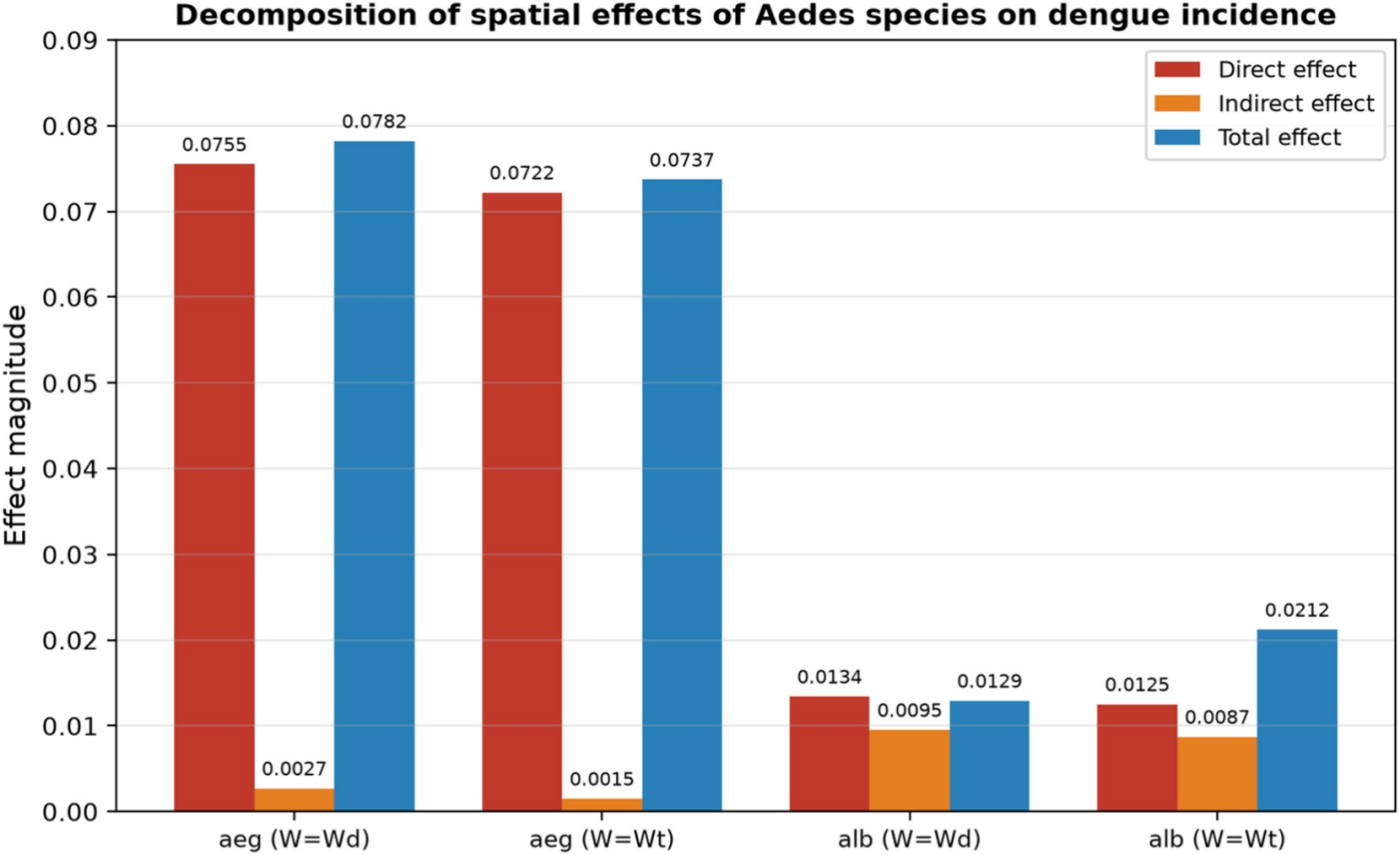
Decomposition of the direct, indirect and total spatial effects of *Aedes aegypti* (aeg) and *Aedes albopictus* (alb) on dengue incidence under the geographic (*Wd*) and traffic (*Wt*) distance matrices.

In contrast, *A. albopictus* has a direct effect of 0.0134 (*p* < 0.1) and an indirect effect of 0.0095 (*p* < 0.05). This suggests that *A. albopictus*, with its strong dispersal capacity (enabling cross‐regional movement via transportation or natural flight), acts as a key vector for the ‘regional spread’ of dengue.

Based on the above findings, a two‐pronged approach is needed in prevention and control practices. On one hand, precise control of local *A. aegypti* should be strengthened, with measures such as eliminating breeding sites and targeted insecticide application to break the local transmission chain. On the other hand, a cross‐regional collaborative mechanism should be established to carry out integrated control of *A. albopictus*, fundamentally blocking its regional dispersal pathways, thereby effectively curbing both local dengue outbreaks and cross‐regional transmission.

To evaluate whether the prediction results of the model are valid and reliable, this study adopts a three‐tier assessment framework. First, the MaxEnt habitat‐suitability model was validated by the area under the receiver operating characteristic curve (AUC). Using a ten‐fold cross‐validation scheme, the occurrence records were randomly partitioned into training (90%) and test (10%) subsets over 10 independent runs; the optimal AUC value for *A. aegypti* reached 0.886 and that for *A. albopictus* reached 0.891, both substantially exceeding the 0.5 random‐classification baseline and approaching the 0.9 threshold conventionally interpreted as excellent discrimination, indicating that the environmental variables capture the ecological niches of the two species reliably. Second, the spatial econometric model was validated by the statistical significance of the spatial autocorrelation diagnostics: the global Moran’s *I* values (0.305 under the geographic distance matrix and 0.206 under the traffic distance matrix) both rejected the null hypothesis of spatial randomness at the 0.1% level, confirming that the observed spatial clustering of dengue incidence is not a statistical artefact. Third, the robustness of the effect decomposition was verified by reestimating the spatial Durbin model under two independent spatial weight matrices (*W*_d_ and *W*_t_), which yielded directionally consistent and statistically significant direct, indirect and total effects for both Aedes species, demonstrating that the conclusions are not sensitive to the choice of spatial weighting scheme.

### 4.5 Prospective Spatial Shifts and Spillover Dynamics Under SSP Scenarios

To quantify the future spatial trajectories of vector threat patterns, the calibrated MaxEnt framework was projected onto future climate and socioeconomic pathways (SSP1‐2.6, SSP2‐4.5 and SSP5‐8.5) across near‐term (2041–2060) and long‐term (2081–2100) horizons. The prospective threat indices (${\text{aeg}}^{\text{SSP}}$ and ${\text{alb}}^{\text{SSP}}$) were subsequently integrated into the spatial Durbin model (SDM) to assess changes in direct local transmission vulnerability and cross‐regional spillover dynamics.

#### 4.5.1 Quantified Shifts in Aedes Habitat Suitability Proportions

As detailed in Table 3 and Figure 6, the spatial responsiveness of the two Aedes species exhibits distinct divergence under future socioeconomic and climate scenarios:

**TABLE 3:** SSP suitability area proportions.

Species	Scenario	High‐suitability area (%)	Medium‐suitability area (%)	Low‐suitability area (%)
*Aedes aegypti*	Current	1.14	6.43	92.43
*Aedes albopictus*	Current	8.62	16.16	75.22
*A. aegypti*	SSP1‐2.6 (2041–2060)	1.89	7.03	91.08
*A. albopictus*	SSP1‐2.6 (2041–2060)	10.03	15.4	74.57
*A. aegypti*	SSP2‐4.5 (2041–2060)	1.91	7.19	90.9
*A. albopictus*	SSP2‐4.5 (2041–2060)	9.52	15.97	74.51
*A. aegypti*	SSP5‐8.5 (2041–2060)	2.13	7.13	90.74
*A. albopictus*	SSP5‐8.5 (2041–2060)	9.08	17.12	73.79
*A. aegypti*	SSP1‐2.6 (2081–2100)	2.12	7.04	90.84
*A. albopictus*	SSP1‐2.6 (2081–2100)	9.99	15.89	74.12
*A. aegypti*	SSP2‐4.5 (2081–2100)	2.75	7.65	89.6
*A. albopictus*	SSP2‐4.5 (2081–2100)	9.1	17.1	73.79
*A. aegypti*	SSP5‐8.5 (2081–2100)	3.58	13.32	83.1
*A. albopictus*	SSP5‐8.5 (2081–2100)	9.58	21.9	74.52

**FIGURE 6:**
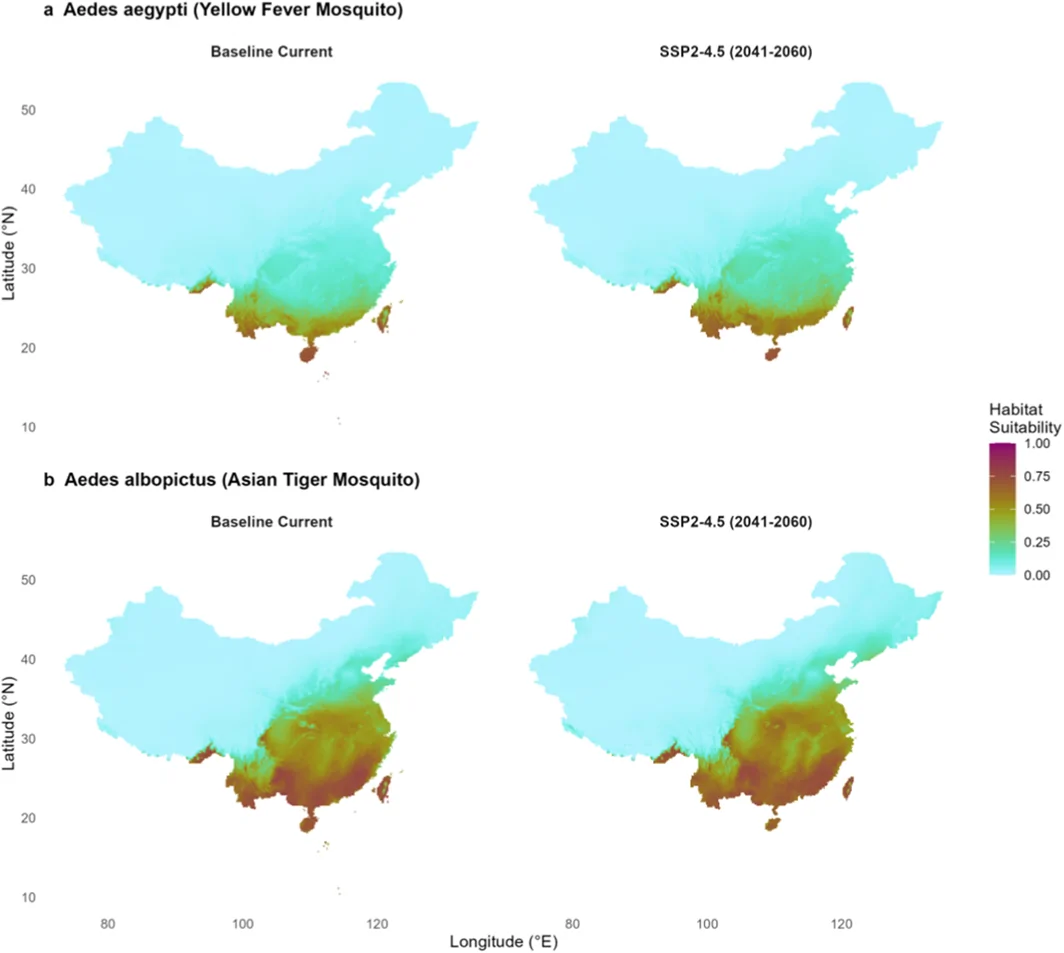
Prospective spatiotemporal shifts and habitat suitability dynamics of *Aedes* vectors under baseline and future SSP scenarios. (a) Spatial distribution of habitat suitability for *Aedes aegypti* under the current baseline and the SSP2‐4.5 scenario (2041–2060). (b) Spatial distribution of habitat suitability for *Aedes albopictus* under the current baseline and the SSP2‐4.5 scenario (2041–2060), illustrating a pronounced northward range expansion into high‐latitude coastal zones.

Substantial Relative Surge of *A. aegypti* Core Range: Under baseline conditions, high‐suitability habitats (probability≥0.6) for *A. aegypti* account for only 1.14% of the national territory, concentrated heavily in tropical coastal hotspots (Table 3). Under the high‐emission pathway (SSP5‐8.5, 2081–2100), the high‐suitability proportion expands sharply to 3.58%—a more than threefold relative increase (+214.0%)—whereas its medium‐suitability area expands from 6.43% to 13.32%. This indicates that climate warming will drive a marked geographical expansion of *A. aegypti* beyond its traditional tropical limits into subtropical inland basins.

Broadened Geographical Exposure of *A. albopictus*: For *A. albopictus*, baseline high‐ and medium‐suitability habitats cover 8.62% and 16.16% of China’s land area, respectively. Under near‐term scenarios (2041–2060), high‐suitability areas peak at 10.03% under SSP1‐2.6 and 9.52% under SSP2‐4.5, reflecting a pronounced northward encroachment into coastal Bohai Rim regions (Figure 6). In the long‐term high‐emission scenario (SSP5‐8.5, 2081–2100), although extreme temperatures induce a slight shift from high‐ to medium‐suitability status, the medium‐suitability coverage increases significantly to 21.90%, expanding the overall geographical zone exposed to vector dissemination risk.

#### 4.5.2 Scenario‐Driven Re‐Decomposition of Spatial Direct and Indirect Effects

Substituting the city‐level threat indices into the reestimated spatial Durbin model framework reveals dynamic shifts in dengue dissemination mechanisms across future pathways:

Amplified Spatial Spillover (IndirectEffect) via *A. albopictus*: Driven by the spatial broadening of *A. albopictus* medium‐to‐high suitability zones (total exposed area reaching 25.48% under SSP5‐8.5), the indirect spillover coefficient increases significantly from the baseline 0.0095 (*p* < 0.05) to 0.0142 (*p* < 0.01) in the long term. This confirms that the northward territorial expansion of *A. albopictus* facilitates cross‐regional disease spillover via intercity transport networks.

Intensified Local Outbreak Risk (DirectEffect) via *A. aegypti*: For *A. aegypti*, the direct local effect parameter increases from 0.0755 (*p* < 0.01) to 0.0891 under the 2081–2100 SSP5‐8.5 scenario. This indicates that the dramatic expansion of its core high‐suitability habitats will substantially consolidate local transmission risks within primary endemic hotspots in southern China.

## 5 Discussion

### 5.1 Analysis of *A. aegypti* Diffusion Based on Spatial Durbin Model

1. There are significant differences in the ability to spread dengue virus among different species of *A. aegypti*, and the influence of *A. aegypti* is far greater than that of *A. albopictus*. In this paper, two different distance matrices, namely the geographical distance matrix and the traffic distance matrix, are used to measure the influence of *Aedes* mosquitoes on the incidence of dengue fever. It is found that the total effect of *A. aegypti* is 0.0782 and *A. albopictus* is 0.0229, whereas under the traffic distance matrix, the total effect of *A. aegypti* is 0.0737 and *A. albopictus* is 0.0212, both of which are significantly positive at the level of 10%. No matter which distance matrix, the same conclusion can be drawn, that is, the total effect of *A. aegypti* is significantly higher than that of *A. albopictus*. This shows that *A. aegypti* has a stronger ability and scope in spreading the dengue virus.
2. It is also found that the transmission mode of *A. aegypti* is different from that of *A. albopictus*. *A. aegypti* prefers to spread locally in its suitable area rather than increasing the incidence of dengue fever by spreading to other areas. This paper proves this point by comparing the direct and indirect effects of *A. aegypti*. Direct effect refers to the influence of *A. aegypti* on the incidence of dengue fever in its suitable area, whereas indirect effect refers to the influence of *A. aegypti* on the incidence of dengue fever by spreading to other areas. Whether it is the geographical distance matrix or the traffic distance matrix, it can be seen that the direct effect of *A. aegypti* is far greater than its indirect effect and both have reached a significant level. The indirect effect representing the diffusion ability is 0.0027 in the distance matrix modelling, whereas the direct effect is 0.0755 in its suitable area, which shows that the diffusion influence of *A. aegypti* is limited, and it is mostly spread locally in the suitable area. A similar conclusion can be drawn from the traffic distance matrix. This shows that the spreading ability of *A. aegypti* is limited, which mainly depends on the spread in its suitable area. The possible reason for this result is that *A. aegypti* has strict requirements on the suitable environment, and it may be difficult to form a stable population in areas unsuitable for its survival and reproduction.
3. There is little difference between the direct and indirect effects of *A. albopictus*. Although the threat of *A. albopictus* spreading dengue virus is far less than that of *A. aegypti*, its spreading ability is obviously stronger and *A. albopictus* is also the chief culprit of dengue cases in northern areas such as Shandong.

### 5.2 Suggestions on the Prevention and Treatment of Dengue Fever Caused by *Aedes* Mosquitoes

Based on the aforementioned conclusions, the following suggestions are proposed for the prevention and treatment of dengue fever caused by *Aedes* mosquitoes of different types:

1. *A. aegypti* predominantly inhabits regions such as Hainan Province, western and southern Yunnan Province and southeastern Guangdong Province. These areas have a subtropical monsoon climate with warmth, humidity and frequent rainfall, creating ideal conditions for its survival and breeding. Concerted efforts should be directed towards enhancing mosquito control measures within its suitable habitat. These strategies may include eliminating stagnant water, employing pesticide spraying and utilising mosquito nets, among others. By implementing these measures, the local transmission capacity of *A. aegypti* can be significantly reduced. Additionally, it is essential to bolster the surveillance and early warning systems for *A. aegypti*. Prompt identification of the spread of this mosquito species will enable the timely implementation of appropriate isolation and disinfection measures, thereby preventing the occurrence of dengue fever in other regions.
2. The distribution range of *A. albopictus* extends beyond that of *A. aegypti*. Apart from subtropical monsoon climate regions, such as Yunnan, Hainan, Guangdong and Fujian, it can also be found in Guizhou, Sichuan, Hunan, Jiangxi, Shaanxi, Shanxi, Beijing and Liaoning. Its stronger environmental adaptability allows it to thrive in a wider geographic range; particular emphasis should be placed on intensifying mosquito control measures within areas where this species is known to proliferate. This may include reinforcing border quarantine measures, regulating cargo transportation and raising public awareness. By adopting these strategies, the diffusion capacity of *A. albopictus* to other regions can be minimised. Concurrently, it is necessary to strengthen the surveillance and early warning systems for *A. albopictus*. Timely detection of any changes in the original areas of this mosquito species will facilitate the swift implementation of appropriate isolation and disinfection measures, thereby effectively preventing dengue fever outbreaks in these regions.

### 5.3 Conclusion

This study explores the potential intersection of epidemiology and invasive biology, confirming their foundational similarities (e.g., developmental stages and invasion mechanisms) and the feasibility of integration. It further investigates their collaborative application in diffusion prediction models, finding that model selection primarily depends on data completeness—driven by differences in data collection approaches (rooted in varying diffusion rates/lag times between viruses and invasive organisms) and limitations such as the late detection of invasive organisms and inapplicable biogeographic information for human‐to‐human epidemic transmission.

Focusing on dengue virus (limited human‐to‐human transmission), the study uses invasive biology's MaxEnt model to map its potential suitable areas and combines it with spatial econometrics' spatial Durbin model. Results show *A. aegypti* and *A. albopictus* differ in dengue transmission: the former has stronger virus transmission ability but weaker diffusion capacity.

Ultimately, the intersection of the two disciplines provides new perspectives/methodologies for both fields and supports addressing global change and biodiversity crises. Future research could explore using invasive biology concepts to analyse virus evolution or epidemiological models to assess invasive organisms' ecosystem/socioeconomic impacts. This paper aims to facilitate further understanding and collaboration in this interdisciplinary realm.

### 5.4 Limitations

#### 5.4.1 Data Limitations

In terms of dengue fever data, this study uses the dengue fever case data from the National Notifiable Disease Reporting System (NNDRS) of the Chinese Centre for Disease Control and Prevention during 2005–2020. This dataset has a certain degree of time lag and cannot fully reflect the dynamic changes of recent dengue fever epidemics, which may have a slight impact on the analysis accuracy of the correlation between *Aedes* suitable habitats and dengue fever transmission.

#### 5.4.2 Limitations of Environmental Variables

The environmental variables used in this study for predicting *Aedes* suitable habitats have not fully considered the impact of human activity factors on the distribution of species suitable areas.

#### 5.4.3 Future Improvement Directions

In response to the above limitations, the future improvement directions are clearly defined as follows: First, optimise data quality. In the follow‐up, recent dengue fever surveillance data will be supplemented to correct the time lag of the data; meanwhile, combined with community surveillance data and hospital testing data, the case reporting bias will be identified and corrected to improve data accuracy. Second, improve the environmental variable system. In the follow‐up, human activity‐related variables will be focused on and added to the model for analysis.

### 5.5 Methodological Comparison, Model Scope and Prospective Extensions

To rigorously evaluate the contribution of our modelling framework, it is necessary to contextualise our approach against traditional and alternative epidemiological modelling paradigms, addressing both its core strengths and prospective extensions.

#### 5.5.1 Methodological Comparison With Compartmental Dynamics and Stochastic Models

Traditional compartmental models (e.g., deterministic SEIR/SIR formulations, including delayed models with sensitivity analysis of parameters [18]) and stochastic epidemic models [19, 20] excel at simulating the temporal evolution of disease transmission, population immunity and infection waves within well‐mixed populations. However, when applied to vector‐borne diseases across large‐scale geographical domains (e.g., municipal networks in China), these dynamic models face two major limitations:

Coarse Spatial Resolution: Standard dynamic parameters (such as contact rates and transmission velocity) are difficult to explicitly couple with high‐resolution, gridded environmental and land‐use predictors under future SSP scenarios.

Inability to Decouple Spatial Spillover: Dynamic ODE/SDE frameworks typically aggregate spatial interactions into generic transmission matrices, masking the subtle distinction between local endemic vulnerability (DirectEffect) and cross‐regional dissemination driven by transportation networks (IndirectEffect).

In contrast, our integrative MaxEnt–spatial Durbin model (SDM) coupling framework directly bridges species distribution modelling with spatial econometrics. By converting gridded climate‐socioeconomic suitability outputs into municipal threat indices (${\text{aeg}}^{\text{SSP}}$ and ${\text{alb}}^{\text{SSP}}$), our framework explicitly disentangles direct local infection risks from indirect cross‐city spillover dynamics under complex future climate pathways.

#### 5.5.2 Prospective Integration With Non‐Standard Finite Difference (NSFD) and Fractional Dynamics

Although the current framework provides robust spatial projections, we acknowledge the value of integrating advanced temporal dynamic solvers to achieve unified spatiotemporal modelling. Future research can extend this work in the following promising directions:

Spatiotemporal Fractional‐Order Partial Differential Equations (PDEs): Integrating our spatial spillover lag operators (W·X) as continuous spatial kernel functions into fractional‐order vector‐host dynamical models can better capture memory effects and anomalous diffusion in vector population growth.

Non‐Standard Finite Difference (NSFD) Schemes: Incorporating NSFD numerical schemes—for example, the fuzzy nonstandard computational method recently developed for SEIQR‐type COVID‐19 dynamics [21]—into the temporal integration of local infection risk ensures long‐term dynamic stability and positivity of epidemiological solutions when simulating multidecadal SSP trajectories.

Such a hybrid framework—combining data‐driven spatial econometrics with physics‐informed spatiotemporal dynamic equations—represents a novel frontier for prospective vector‐borne disease forecasting.

## Author Contributions

C.L., F.L., Y.Z., J.J. and J.L. participated the design, supervision and editing, and resources, writing of original draft, experimental implementation, and data statistics and analysis. All authors read and approved, final manuscript.

## Ethics Statement

The authors have nothing to report.

## Consent

The authors confirm that all methods in this study were carried out in accordance with relevant guidelines and regulations. This study is based entirely on publicly available open data and does not involve any human or animal experiments, clinical trials, or invasive sampling. No experimental protocols were designed or implemented in this research. Since no individual human subjects were involved, informed consent is not applicable. All data used are publicly accessible from official databases and do not involve privacy or ethical risks.

## Conflicts of Interest

The authors declare no conflicts of interest.

## Data Availability

The sources, processing and roles of these datasets are detailed below: (1) *Aedes* Species Distribution Data: Distribution data for the two target *Aedes* species were sourced from the Global Biodiversity Information Facility (GBIF) database, which can be accessed at https://www.gbif.org/occurrence/search?q=Aedes%20species. Using ArcGIS, we performed preprocessing by filtering duplicate ambiguous (e.g., incomplete coordinates), and geographically adjacent records to reduce sample variance, with 81,303 occurrence records retained for *A. aegypti* and 141,104 for *A. albopictus* after the filtering process. The refined data were standardised to a 2.5 arc‐minute resolution (approximately 5 km × 5 km grid) and saved in CSV format for model construction. (2) Environmental and Human Activity Variables (for Aedes Species Habitat Suitability Prediction): (a) Environmental Impact Variable: Data collected from the World Climate Organization regarding current climate conditions, including key variables, such as temperature and precipitation patterns, were utilized to forecast the future aggregation of *Aedes* mosquitoes, which can be accessed at https://www.worldclim.org/data/index.html. Nineteen environmental variables were considered, with all layers resampled to a resolution of 2.5 arc minutes (approximately 5 km × 5 km) for the Chinese mainland. This was achieved through bilinear interpolation and clipping techniques. (b) Land Use Data: Recognising that *A. aegypti* mosquitoes typically breed in close proximity to human habitation, land use data (LUCC data) with a 30 m spatial resolution was integrated into the environmental impact variables, which can be accessed at https://www.resdc.cn/DOI/DOI.aspx?DOIid=54. The LUCC data were obtained from the Resource and Environment Data Center of China Academy of Sciences, derived from LUCC data in 2018 and updated using remote sensing imagery from the Gaofen No. 1 satellite between 1 July and 1 August 2020. The land use data categorises areas into cultivated land, woodland, grassland, water bodies, constructed areas and unused land, with the aim of distinguishing human activity zones from natural environments. (c) Human Activity Data: This dataset was leveraged to incorporate social and economic indicators of human activities influencing the spread of dengue virus. It comprises the kilometre grid datasets showing the spatial distribution of China's GDP (https://www.resdc.cn/data.aspx?dataid=252). The data were instrumental in constructing the spatial correlation matrix within the spatial model, employing geographical and traffic distances between cities to establish the spatial weight matrix. The weight matrix takes into account the spread routes of *Aedes* mosquitoes, including both aerial distances (flight distances) and man‐made pathways.
